# Phylogenomic Analysis Reconstructed the Order Matoniales from Paleopolyploidy Veil

**DOI:** 10.3390/plants11121529

**Published:** 2022-06-07

**Authors:** Jiang-Ping Shu, Hao Wang, Hui Shen, Rui-Jiang Wang, Qiang Fu, Yong-Dong Wang, Yuan-Nian Jiao, Yue-Hong Yan

**Affiliations:** 1 Shenzhen Key Laboratory for Orchid Conservation and Utilization, and Key Laboratory of National Forestry and Grassland Administration for Orchid Conservation and Utilization, the National Orchid Conservation Center of China and the Orchid Conservation & Research Center of Shenzhen, Shenzhen 518114, China; jpshu@scbg.ac.cn; 2 Key Laboratory of Plant Resources Conservation and Sustainable Utilization, South China Botanical Garden, Chinese Academy of Sciences, Guangzhou 510650, China; wangrj@scbg.ac.cn; 3 Shanghai Chenshan Plant Science Research Center, Shanghai Chenshan Botanical Garden, Chinese Academy of Sciences, Shanghai 201602, China; wanghao6081336@163.com (H.W.); shenhui@cemps.ac.cn (H.S.); 4 State Key Laboratory of Palaeobiology and Stratigraphy, Nanjing Institute of Geology and Palaeontology, and Center for Excellence in Life and Paleoenvironment, Chinese Academy of Sciences, Nanjing 210008, China; jinhua@nigpas.ac.cn (Q.F.); ydwang@nigpas.ac.cn (Y.-D.W.); 5 Institute of Botany, The Chinese Academy of Sciences, Beijing 100039, China; jiaoyn@ibcas.ac.cn

**Keywords:** ancient polyploidization, ancient radiation, reticulate evolution, mass extinction, ferns, phylogeny, 1-to-1 orthologous genes

## Abstract

Phylogenetic conflicts limit our understanding of the evolution of terrestrial life under multiple whole genome duplication events, and the phylogeny of early terrestrial plants remains full of controversy. Although much incongruence has been solved with so-called robust topology based on single or lower copy genes, the evolutionary mechanisms behind phylogenetic conflicts such as polyploidization remain poorly understood. Here, through decreasing the effects of polyploidization and increasing the samples of species, which represent all four orders and eight families that comprise early leptosporangiate ferns, we have reconstructed a robust phylogenetic tree and network with 1125 1-to-1 orthologs based on both coalescent and concatenation methods. Our data consistently suggest that Matoniales, as a monophyletic lineage including Matoniaceae and Dipteridaceae, should be redefined as an ordinal rank. Furthermore, we have identified and located at least 11 whole-genome duplication events within the evolutionary history of four leptosporangiates lineages, and associated polyploidization with higher speciation rates and mass extinction events. We hypothesize that paleopolyploidization may have enabled leptosporangiate ferns to survive during mass extinction events at the end Permian period and then flourish throughout the Mesozoic era, which is supported by extensive fossil records. Our results highlight how ancient polyploidy can result in rapid species radiation, thus causing phylogenetic conflicts yet allowing plants to survive and thrive during mass extinction events.

## 1. Introduction

The tree of life has been one of the biggest challenges to biologists since Darwin; phylogenetic tree reconstruction vastly improves our understanding of systematic taxonomy and the evolution of terrestrial life [1,2], which includes prokaryotes [3] and eukaryotes [4] and sub-classifications such as animals [5], insects [6], fungi [7], and plants [8,9,10]. However, with continuing phylogenetic research, more and more discordances come to us [11], even in deep phylogenetic relationships. In general, new studies include only a brief description of the robustness of their results with high bootstrap values and/or posterior probability and report differences with other studies; however, the evolutionary mechanisms underlying these phylogenetic conflicts are rarely discussed.

Polyploidization, including autopolyploidization, such as whole-genome duplication (WGD), and allopolyploidization, such as hybrid polyploidization, is an important evolutionary force and a driver of plant speciation [12,13]. WGD and reticulate evolution, which are prevalent in vascular plants, especially in ferns [10,14,15,16], are considered as the major factors affecting the stability and reliability of the phylogenetic relationship [17,18]. Therefore, ancient polyploidy events may underlie some phylogenetic conflicts, especially those in deep phylogenetic relationships; however, this hypothesis currently lacks veritable evidence. The remaining challenge is how to reconstruct the clear phylogenetic relationship under multiple WGDs.

**Figure 1:**
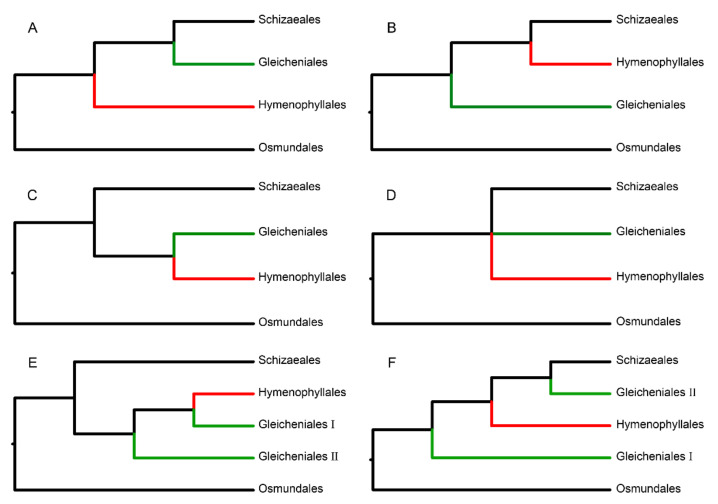
Six topologies of the phylogenetic relationship in the early leptosporangiate ferns. The corresponding references are shown in Table S1. The red branches indicate Hymenophyllales, and the green branches indicate Gleicheniales. Gleicheniales I indicates Gleicheniaceae, and Gleicheniales II indicates Dipteridaceae and Matoniaceae.

Previous studies have remarkably improved our understanding of the evolutionary history of ferns [19,20,21,22,23,24,25]. Nevertheless, phylogenetic conflicts remained in the leptosporangiate ferns, which contained seven orders, i.e., Osmundales, Hymenophyllales, Gleicheniales, Schizaeales, Salviniales, Cyatheales, and Polypodiales [20]. To date, there are at least six phylogenetic topologies between Hymenophyllales and Gleicheniales constructed by previous studies (Figure 1, Table S1). In general, Gleicheniales is considered to be a monophyletic lineage that includes Dipteridaceae, Matoniaceae, and Gleicheniaceae; this lineage is a sister group of the remaining leptosporangiate ferns except for Osmundales and Hymenophyllales (Figure 1a) [23,24,25,26,27,28]. Interestingly, Pryer et al., (2004) proposed a sistership between gleichenioid and filmy ferns (Figure 1c), which was supported by molecular, morphological, and fossil evidence. However, these studies mostly relied on a few plastid genes or those combined with nuclear genes and/or morphological traits, which may not be sufficient to resolve deep phylogenetic relationships [2].

Following the development of next-generation sequencing and phylogenomic analysis, Shen et al., (2018) and Qi et al., (2018) reconstructed the framework of fern phylogeny based on massive transcriptomic datasets with 69 and 129 samples, respectively. This revealed that gleichenioid ferns are not a monophyletic group and that Gleicheniaceae is a sister group of Hymenophyllaceae (Figure 1e), which means that Dipteridaceae might be another order of Dipteridales [29]. However, Matoniaceae, belonging to Matoniales [30] and considered as the sister lineage of Dipteridaceae [25,28], was not included in these two studies. Taxon sampling, which is considered as a common pitfall in phylogenetics [31,32], plays an important role in phylogenetic inference accuracy [5,33,34,35], and thus it is expected that a degree of the sampling within a study can be compromised by systematic errors [36]. Here, in order to increase taxa coverage, we analyzed the transcriptome data of 31 ferns, including 21 newly sequenced species. These data represent all of the four orders (Osmundales, Hymenophyllales, Gleicheniales, and Schizaeales) and eight families (Osmundaceae, Hymenophyllaceae, Matoniaceae, Dipteridaceae, Gleicheniaceae, Lygodiaceae, Schizaeaceae, and Anemiaceae) that comprise early leptosporangiate ferns.

It has been proposed that ferns underwent multiple cycles of polyploidization accompanied by subsequent diploidization but without apparent chromosome loss [37]. Moreover, evidence of repeated WGD during the diversification of leptosporangiate ferns was reported [38,39]. Due to the evolutionary significance of polyploidization and its effect on the phylogenetic relationship [13,18], we hypothesized that ancient polyploidization may have occurred in the early leptosporangiate ferns. This may have enabled ferns to survive and flourish during the Mesozoic era, but it has hampered the reconstruction of a clear and stable phylogenetic relationship to date.

In this study, we aimed to perform different analytical strategies to decrease the effect of polyploidization on phylogenomic analysis by 1-to-1 ortholog genes and to reconstruct robust phylogenetic relationships within early leptosporangiate ferns using sufficiently representative taxa. In addition, to improve our understanding of the impact of polyploidization in fern evolution, we investigated whether WGD and reticulate evolution underlie the ambiguous evolutionary history of early leptosporangiate ferns.

## 2. Results

### 2.1. Assessment of RNA-Seq Data

**Figure 2:**
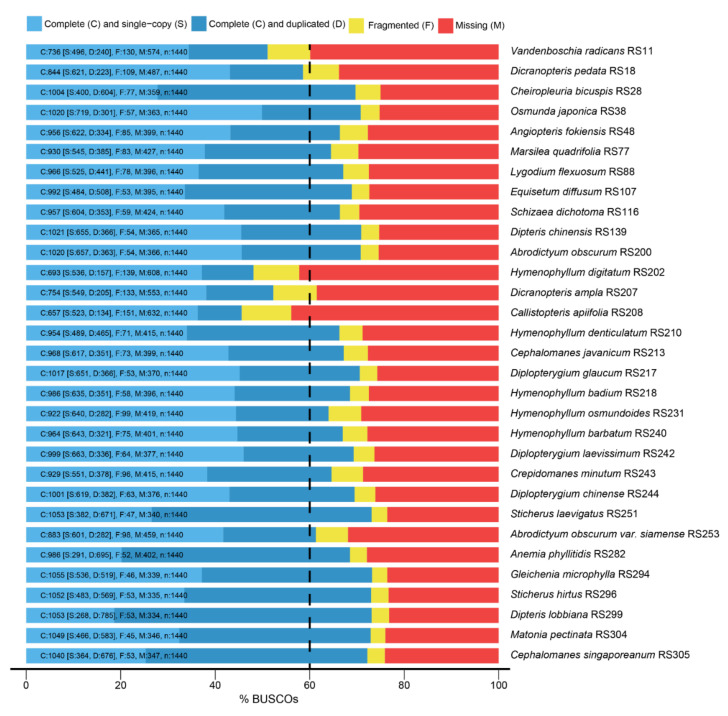
The assessment of transcriptome assembly completeness by Basic Universal Single Copy Orthologs (BUSCOs) analysis. Except for *Hymenophyllum digitatum* (RS202) and *Callistopteris apiifolia* (RS208), transcriptome assemblies of the sampled species were complete and orthologs shared more than 60% sequence identity.

New transcriptomic data were generated from 21 fern species (Table S2, RS200-RS305). After filtering, approximately 1354.3 million total reads (about 200 Gb) were obtained. The average of Q30 and N50 were 94% and 1741 bp, respectively. In order to assess the assembly completeness of our transcriptomic data, we employed the core set of orthologs that are ultra-conserved in embryophyte species, which contained 1440 BUSCOs. Following a BLAST against our transcriptomic data, the gene coverage of all samples used in this study was more than 60%, except for *Hymenophyllum digitatum* (RS202, 57%) and *Callistopteris apiifolia* (RS208, 56%), and at least 808 conservative orthologs were matched (Figure 2).

### 2.2. Evolutionary History of the Early Leptosporangiate Ferns

**Figure 3:**
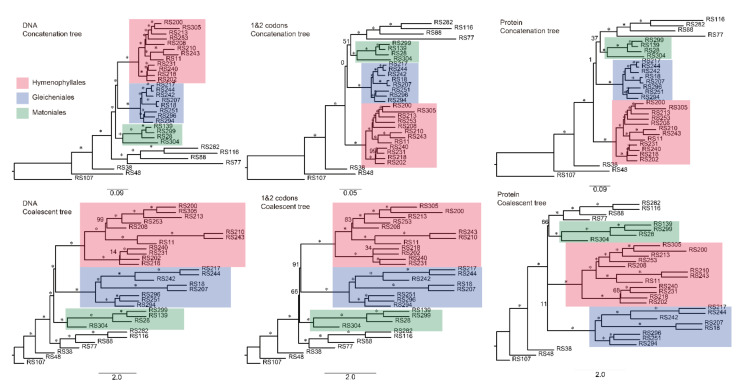
Six phylogenetic trees of different strategies. The number on the branch is the bootstrap value, and the asterisk (*) indicates a bootstrap support (BS) value of 100. The DNA concatenation tree, the DNA coalescent tree, and the 1 and 2 codons coalescent tree are consistent with high confidence (almost all of BS = 100), whereas the other trees are incongruent with low BS values.

**Figure 4:**
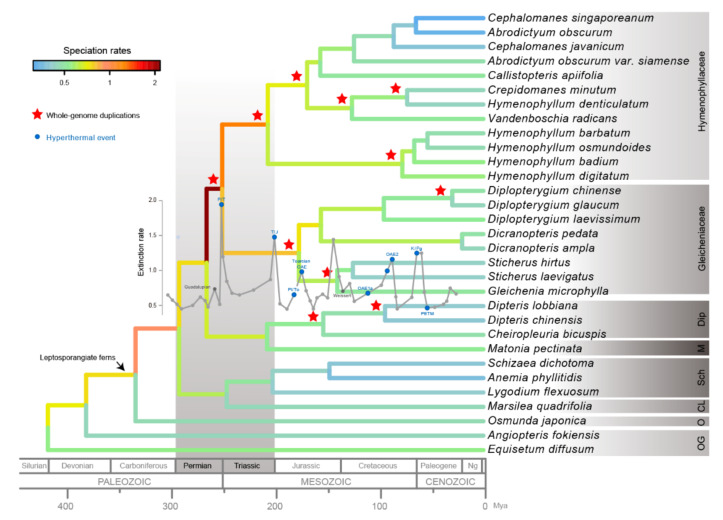
The evolutionary history of early leptosporangiate ferns. The bootstrap support (BS) value of each branch is 100 (not indicated in the species tree). The extinction rate was sourced from Clapham and Renne (2019). The red stars indicate the inferred whole genome duplication events. Abbreviations: K/Pg, end-Cretaceous; OAE, oceanic anoxic event; PETM, Paleocene–Eocene Thermal Maximum; Pl/To, Pliensbachian–Toarcian; P/T, end-Permian; T/J, end-Triassic; Dip, Dipteridaceae; M, Matoniaceae; Sch, Schizaeales; CL, core leptosporangiate; O, Osmundales; and OG, outgroups.

After careful screening using the ortholog-derived pipeline (Figure S1) described in Shen et al., (2018), 1125 1-to-1 orthologs consisting of 878,952 nucleotides, each of which represented at least 16 species, were identified and used in subsequent analyses. We used both coalescent- and concatenation-based methods to infer the phylogenetic relationships of early leptosporangiate ferns (Figure 3). All species trees indicated that Gleicheniaceae is the sister group of Hymenophyllaceae and that sistership also exists between Dipteridaceae and Matoniaceae; however, Gleicheniales, which included Gleicheniaceae, Dipteridaceae, and Matoniaceae, was not a monophyletic group (Figure 3 and Figure 4). As shown by both coalescent and concatenation analyses based on the DNA dataset, these four families are a monophyletic lineage with high confidence (Figure 3 and Figure 4); however, analyses using the other datasets (1 and 2 codons and Protein datasets) showed that these four families are a paraphyletic group with low BS values (Figure 3).

Based on the high-confidence species tree, we determined divergence times in the early leptosporangiate ferns based on a Bayesian relaxed clock model. Our results showed that the leptosporangiate ferns originated in the Carboniferous period about 334 Mya [95% HPD = (261–401 Mya)], and the origins of Gleicheniales and Hymenophyllales were dated to almost the same periods of late Permian and early Triassic (Figure 4 and Figure S2). Following this, early leptosporangiate ferns diversified throughout nearly the entire Mesozoic era. Subsequently, we estimated the species-specific diversification rates based on the reconstructed phylogenetic tree with time estimation. This showed that speciation rates were varied both among tree branches and through time. The highest diversification rate (about 2 Myr^−1^) was calculated in the common ancestor of Hymenophylaceae and Gleicheniaceae, and, generally, speciation rates were more than 0.5 Myr^−1^ in the deep branches of the early leptosporangiate ferns (Figure 4).

### 2.3. Paleopolyploidization and Reticulate Evolution in the Early Leptosporangiate Ferns

**Figure 5:**
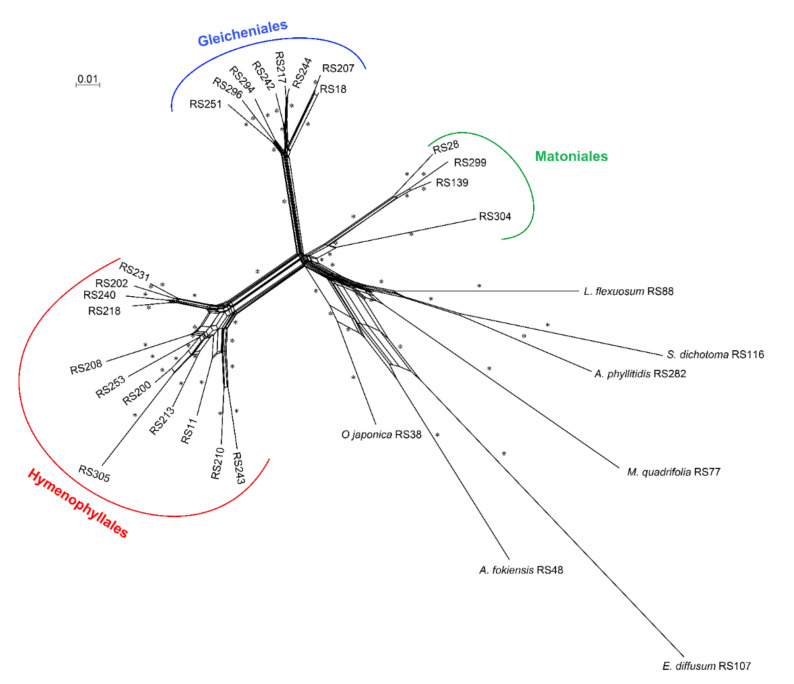
Reticulate evolution in the early leptosporangiate ferns. The asterisk (*) indicates bootstrap support (BS) values of 100.

We applied gene-tree-species tree reconciliation methods to identify and locate WGD events in the early leptosporangiate ferns. After filtering the tandem duplications, at least 11 WGD events were identified during the evolution of early leptosporangiate ferns, especially in the ancestors of Hymenophyllaceae and Gleicheniaceae (Figure 4 and Figure S3). Following this, we reconstructed the phylogenetic network of early leptosporangiate ferns and uncovered a complex evolutionary network with 106 splits and 954 edges. Apparently, the elusive network topology was clearly divided into nine groups with high confidence (Figure 5). Once rooted with *E. diffusum* as the outgroup, the four families (Matoniaceae, Dipteridaceae, Gleicheniaceae, and Hymenophyllaceae) were aggregated as one group. However, the relationships between these families were trifurcate rather than bifurcate due to the presence of hyper-complexed reticulate evolution (Figure S4). Moreover, reticulate evolution was also prevalent in the inner taxa of these families.

## 3. Discussion

### 3.1. Ancient Polyploidization Plays an Important Role in the Evolutionary History of Plants

Polyploidy is significant in many ways in the evolutionary process of organisms [14], and its occurrence is prevalent in vascular plants, especially in ferns [16]. Here, we identified and located WGD events based on the optimal species tree. As expected, at least 11 WGD events were identified during the evolutionary history of early leptosporangiate ferns (Figure 4 and Figure S3). In the literature, it is widely contested how polyploidization affects species diversification [13,40,41]. On the one hand, polyploidization is considered as an evolutionary “dead end” [42,43] that results in a lower diversification rate than that of diploid relatives. There is lacking evidence for WGD events that survive in the long term, and polyploids speciate more slowly and exhibit higher rates of extinction than their diploid progenitors [44]. On the other hand, WGD events often cause higher diversification rates but only after a delay of potentially up to several million years [45,46]. Although we have not carefully investigated the link between species diversification rates and paleopolyploidization, it seems that with the occurrence of more WGD events, there is higher species diversity and a higher diversification rate in clades (Figure 4). This partly indicates that ancient polyploidization, similar to ancient hybridization [47], is a major driver of rapid radiation [17].

In this study, we increased the species representation of early leptosporangiate ferns and used a large-scale transcriptomic dataset including 1125 1-to-1 orthologs; however, the reconstruction of a consistent species tree remained elusive (Figure 3 and Figure 4). Potentially, these topological incongruences are the result of rapid diversification and the complicated phylogenetic network that exists among the four monophyletic lineages Hymenophyllaceae, Gleicheniaceae, Dipteridaceae, and Matoniaceae (Figure 4 and Figure S4), which might also be the result of ancient polyploidization. In other words, the evolutionary histories of many organisms may have an inherent radiate pattern, such as trifurcate (Figure S4), owing to complicated biological processes, such as polyploidization and hybridization [48].

In addition to being a driver of rapid evolution, polyploidization might also lead to increased tolerance to a broader range of ecological and environmental conditions [49,50,51], leading to a better chance of survival during mass extinction events [17,52,53]. Based on our calculated divergence times, the ancestors of Matoniaceae, Dipteridaceae, Gleicheniaceae, and Hymenophyllaceae originated about 266 Ma [95% HPD = (233–351 Ma)] during the late Carboniferous and early Triassic periods (Figure 4) [54]. At that time, the most extensive mass extinction event known to date, referred to as the Permian-Triassic (P-T) event, occurred [55], whereby the eruption of the Siberian Traps exposed an enormous amount of fresh basalt and triggered CO_2_ release, rapid global warming, and acid rain [56]. Regarded as an evolutionary bottleneck, the P-T event narrowed down the gene pool and left a small number of species to repopulate the Earth, upon which there were many empty and new niches. The early leptosporangiate ferns were among those to survive, which then flourished throughout the whole Mesozoic era, especially Dipteridaceae, Matoniaceae, and Gleicheniaceae [54,57,58]. The success of these families may be due to ancient polyploidization (Figure 4). In fact, it seems that polyploidization is strongly correlated with extinction events, in that almost every extinction event is linked to polyploidy events in the evolutionary history of life [13,15]. On the one hand, these polyploidy events may have enabled early leptosporangiate ferns to survive the extinction events. On the other hand, these extinction events may have generated massive vacant ecological niches, thus providing opportunities for heliophytes such as Dipteridaceae and gleichenioid ferns to flourish.

### 3.2. Matoniales Should Be Redefined as a New Ordinal Rank

After increasing the taxa coverage, especially in Matoniaceae, and decreasing the effect of ancient polyploidization, we reconstructed the phylogenetic relationships of the early leptosporangiate ferns with a large-scale transcriptomic dataset. Although phylogenetic conflicts persisted among the four families Hymenophyllaceae, Glecheniaceae, Dipteridaceae, and Matoniaceae following both coalescent- and concatenation-based analysis using different kinds of datasets, Matoniaceae was undoubtedly shown to be a sister group of Dipteridaceae (Figure 3 and Figure 4), which is consistent with previous studies [28,59] and supported by our phylogenetic network (Figure 5 and Figure S4). Furthermore, Gleicheniales was clearly a paraphyletic lineage (Figure 3 and Figure 4), which was also reported by Shen et al., (2018) and Qi et al., (2018). In both our concatenation- and coalescent-based species trees, Matoniaceae and Dipteridaceae, as a monophyly, were clearly paraphyletic to Gleicheniaceae (Figure 4). Thus, these combined results suggest that Matoniaceae and Dipteridaceae should be redefined as a new order.

**Figure 6:**
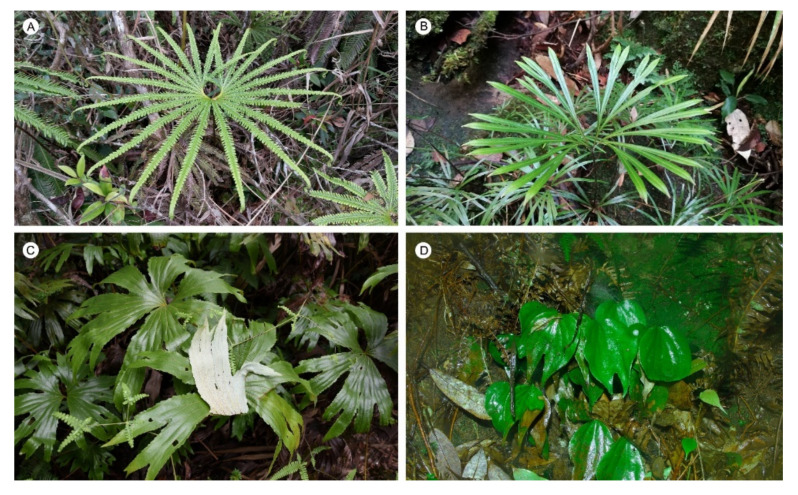
The habit of species from Matoniaceae and Dipteridaceae. (**A**): *Matonia pectinata*; (**B**): *Dipteris lobbiana*; (**C**): *Dipteris chinensis*; and (**D**): *Cheiropleuria bicuspis*.

For a long time, pteridologists have recognized significant differences among the Matoniaceae, Dipteridaceae, and Gleicheniaceae [60], so that Matoniaceae and Dipteridaceae have been treated into the two orders, Matoniales [30] and Dipteridales [29], respectively. Soon after, Mationaceae and Dipteridaceae were merged into Gleicheniales along with Gleicheniaceae, based on monophyletic evidence [28,59,61] and some morphological traits such as root steles with 3–5 protoxylem poles [62] and antheridia with 6–12 narrow, twisted, or curved cells in walls [63], which usually are called gleichids because of common traits of forking branches. In fact, these traits are very common among early leptosporangiate ferns, such as *Schizaea dichotoma* (Schizaeaceae), *Lygodium digitatum* (Lygodiaceae), *Regnellidium diphyllum* (Marsileaceae), and *Gonocormus minutus* (Hymenophyllaceae), and therefore do not provide the classification significance for high order rank. Furthermore, this previous classification is challenged by both our results and those of previous phylogenomic studies [19,22], which supported that Matoniaceae and Dipteridaceae are a monophyletic group but are paraphyletic with Gleicheniaceae, so they should not be merged into the order Gleicheniales. Morphologically, the species in these two families share a number of common morphological characters, such as a creeping rhizome, dichotomous or fan-like split leaves (Figure 6), and flattened sporangia with slightly oblique or sub-erected annulus [64]; they are more or less similar in their spore morphologies (sub-triangular or elliptisoidal spore shape with trilete marks or monolete) [65], and they are mainly distributed in tropical regions in Asia, extending to China and Japan [60,66,67]. Moreover, the sister group of Gleicheniales and Hymenophyllales shared some common sporangia located on the elongated receptacles, with equatorial transverse-oblique annulus and not interrupted by very short stalk, although they look very different in habit [59,60,64].

Based on this robust evidence from phylogenomics and morphology, we propose to redefine a general ordinal rank of Matoniales from Dipteridales Doweld (2001) and Matoniales Reveal (1993) based on the International Code of Nomenclature for algae, fungi, and plants (Shenzhen Code) [68]. This order should be composed of extant Dipteridaceae (including *Cheiropleuria* and *Dipteris*) and Matoniaceae (*Matonia* and *Phanerosorus*), with a total of 15 species [20,63], which flourished with widespread global distribution throughout the Mesozoic era together with their related group of Gleicheniaceae [54,69,70,71]. Certainly, the redefinition of order Matoniales will provide new insights regarding the diversification and prosperity of fossil genera of Matoniaceae and Dipteridaceae, such as *Clathropteris*, *Dictyophyllum*, *Hausmannia*, *Goeppeetella*, *Camptopteris*, *Thaumatopteris*, *Phlebopteris*, *Matonidium*, *Weichselia,* and so on [57,69,72,73,74,75].

An order is a higher taxonomic rank in biological classification, and most of the accepted orders in extant land plants are long-established [20,76]. Only a few new orders of land organisms, such as Mantophasmatodea [6,77] and Hypopterygiales [9], have been recently published and accepted based on phylogenomic evidence. In the extant lycopods and ferns, more than 40 valid orders were reported, but only 14 of these are accepted in PPG I [20,63]. However, more careful studies may be required to verify the treatments of these synonym names in the future [78]. In this study, as an example, we have reconstructed the new order Matoniales from the synonym names with phylogenomic analysis, which was opposed to previous treatments.

## 4. Materials and Methods

### 4.1. Taxon Sampling

Taxon sampling bias was considered as an important factor affecting the phylogenetic relationship [36]. According to PPG I, gleichenioid ferns include approximately 172 species in 10 genera in the three families Dipteridaceae, Matoniaceae, and Gleicheniaceae [20]. In this study, we collected samples of 12 species of gleichenioid ferns in 7 genera representing the 3 families, in particular Matoniaceae, and 12 species of filmy ferns in seven genera. For other early leptosporangiate ferns, we used published data, including those for Osmunda japonica (Osmundaceae), *Lygodium flexuosum* (Lygodiaceae), *Schizaea dichotoma* (Schizaeaceae), and *Anemia phyllitidis* (Anemiaceae), the latter of which was a newly sequenced species. In addition, *Equisetum diffusum* (Equisetaceae) and *Angiopteris fokiensis* (Marattiaceae) as the outgroups and *Marsilea quadrifolia* (Marsilieaceae) as a proxy of core leptosporangiate ferns were used in this study. All samples were collected with permission from the natural reserves of Shanghai Chenshan Botanical Garden in China, and the voucher specimens are kept in the Shanghai Chenshan Herbarium (CSH). The information of materials is shown in Table S2.

### 4.2. Data Acquisition and Quality Assessment

We performed RNA extraction, sequencing, and transcriptome assembly as per in our previous study [19]. In order to assess the gene coverage of each assembly, we performed Basic Universal Single Copy Orthologs (BUSCOs) analysis using a core set of orthologs conserved in Embryophyta species (Embryophyta odb9) [79]. The description of transcriptome data was shown in Table S2.

### 4.3. Phylogenetic Tree and Network Analysis

In order to decrease the effect of paralogs on phylogenetic analysis [80], 1-to-1 orthologs prediction (also illustrated in Figure S1) was in compliance with Shen et al., (2018), but we restricted at least 16 (51.6%) species in each 1-to-1 orthologs matrix, which could decrease the effect of polyploidization on phylogenomic reconstruction [81]. We performed the phylogenomic analysis based on both the coalescent and concatenation methods using three kinds of datasets, including DNA, protein, and especially the first two codons, because saturated third codon positions might radically influence the reconstruction of phylogenetic trees under multiple WGDs [82,83]. In coalescent-based analysis, each gene tree was constructed by RAxML v8.2.4 [84] with the PROTCAT JTTF and GTR model for protein and DNA sequences, respectively; 100 random replicates were performed to calculate bootstrap probabilities. The coalescent-based species tree was constructed by ASTRAL v4.10.4 [85] with 100 random replicates of multilocus bootstrapping [86]. In concatenation-based analysis, a multiple-gene supermatrix was constructed by concatenating the 1-to-1 orthologs matrix, and we created the maximum likelihood (ML) tree by RAxML v8.2.4, with the GTR + Γ4 + I model for the DNA and codon matrices and the JTTF model for the corresponding protein matrix. Following this, tree visualization was performed using FigTree v1.4.3 (http://tree.bio.ed.ac.uk/software/figtree/, accessed on 16 January 2021). Due to numerous complicated evolutionary processes, a phylogenetic network is required in order to graphically represent a reticulate evolutionary history [48]. In this study, we reconstructed the evolutionary network of the early leptosporangiate ferns with SplitsTree4 v4.15.1 [87]. In order to assess the bootstrap support (BS), we performed 1000 random replicates using the Neighbor-Net methods [88] with the Kimura 2-parameter model, and *E. diffusum* was used as the outgroup to infer a rooted phylogenetic network.

### 4.4. Divergence Time Estimation

To date divergence times, we used the concatenated alignment of orthologs and calibrated with the ages of two fossils—*Senftenbergia plumosa* [89]: 354 Ma, and *Hopetedia praetermissa* [90]: 228 Ma—as the minimum age of leptosporangiate ferns and Hymenophyllaceae, respectively, and a maximum age constraint of 450 Ma for monilophytes in a Bayesian relaxed clock method using MCMCTree in PAML v4.9i [91] based on the same robust phylogenetic tree constructed by coalescent and concatenation methods with the DNA and codon matrices.

### 4.5. Polyploidization Inference and Localization

Due to the many limitations of Ks plot analysis [92], especially that it cannot detect more ancient WGD events [93,94,95], which may be prevalent in ferns, we employed a gene-tree sorting and counting algorithm, namely, Multi-tAxon Paleopolyploidy Search (MAPS) [96], to locate WGD events with full gene family dataset, including all paralogs. This algorithm uses a given species tree to filter for subtrees within complex gene trees consistent with relationships at each node in the species tree. WGDs will produce a large burst of shared duplications across taxa and gene trees. We filtered gene trees with less than 16 (51.6%) taxa and defined a WGD as a threshold duplication percentage of more than 10% [15] because lower percentages might result from tandem duplications.

### 4.6. Species Diversification Rates

Understanding how diversification rates vary through time and across species groups is vital to understanding the emergence of the current biodiversity on Earth [97]. However, variation in speciation and extinction rates may exist both across lineages and through time [98]. Here, we used a Bayesian model-based approach, namely, ClaDS of R script (https://github.com/OdileMaliet/ClaDS, accessed on 20 May 2021) [97], to quantify the lineage-specific speciation rates on the basis of our phylogenetic tree with time estimation. The extinction rate data was sourced from Clapham and Renne (2019) with the authors’ permissions.

## 5. Conclusions

To decrease the effect on phylogenetic reconstruction from the WGDs, we have reconstructed the evolutionary history of early leptosporangiate ferns through representative species sampling and analysis of large-scale transcriptomic data by 1-to-1 orthologs. Following careful phylogenomic analysis, we found that the phylogenetic relationships of early leptosporangiate ferns are not bifurcate but rather have a trifurcate pattern due to rapid radiate evolution caused by ancient polyploidization. We propose that Matoniales, which include Dipteridaceae and Matoniaceae, should be redefined as a new ordinal rank based on powerful evidence from 1-to-1 orthologs trees, as with the first two codons. Furthermore, our study found that paleopolyploidization played an important role in the evolutionary history of early leptosporangiate ferns, potentially enabling the survival of these ferns during the P-T mass extinction event and promoting speciation throughout the Mesozoic era.

## Data Availability

The data presented in this study are available on the Genome Sequence Archive (GSA) of National Genomics Data Center (NGDC) with the BioProject number PRJCA009790, and the transcriptome assembly datasets are available on the Figshare database.

## Supplementary Materials

The following supporting information can be downloaded at: https://www.mdpi.com/article/10.3390/plants11121529/s1, Figure S1: The pipeline of 1-to-1 orthologs prediction; Figure S2: The timetree of early leptosporangiate ferns; Figure S3: The inference of WGD events in early leptosporangiate ferns; Figure S4: The rooted reticulate evolution in the early leptosporangiate ferns; Table S1: Summary of main studies of basal leptosporangiate ferns; Table S2: The information of samples and transcriptome data used in this study.

## References

[1] Yang Z., Rannala B. (2012). Molecular phylogenetics: Principles and practice. Nat. Rev. Genet..

[2] Delsuc F., Brinkmann H., Philippe H. (2005). Phylogenomics and the reconstruction of the tree of life. Nat. Rev. Genet..

[3] Fox G.E., Stackebrandt E., Hespell R.B., Gibson J., Maniloff J., Dyer T.A., Wolfe R.S., Balch W.E., Tanner R.S., Magrum L.J. (1980). The phylogeny of prokaryotes. Science.

[4] Baldauf S.L., Roger A.J., Wenk-Siefert I., Doolittle W.F. (2000). A kingdom-level phylogeny of eukaryotes based on combined protein data. Science.

[5] Dunn C.W., Hejnol A., Matus D.Q., Pang K., Browne W.E., Smith S.A., Seaver E., Rouse G.W., Obst M., Edgecombe G.D. (2008). Broad phylogenomic sampling improves resolution of the animal tree of life. Nature.

[6] Misof B., Liu S., Meusemann K., Peters R.S., Donath A., Mayer C., Frandsen P.B., Ware J., Flouri T., Beutel R.G. (2014). Phylogenomics resolves the timing and pattern of insect evolution. Science.

[7] James T.Y., Kauff F., Schoch C.L., Matheny P.B., Hofstetter V., Cox C.J., Celio G., Gueidan C., Fraker E., Miadlikowska J. (2006). Reconstructing the early evolution of Fungi using a six-gene phylogeny. Nature.

[8] Qiu Y.-L., Lee J., Bernasconi-Quadroni F., Soltis D.E., Soltis P.S., Zanis M., Zimmer E.A., Chen Z., Savolainen V., Chase M.W. (1999). The earliest angiosperms: Evidence from mitochondrial, plastid and nuclear genomes. Nature.

[9] Liu Y., Johnson M.G., Cox C.J., Medina R., Devos N., Vanderpoorten A., Hedenäs L., Bell N.E., Shevock J.R., Aguero B. (2019). Resolution of the ordinal phylogeny of mosses using targeted exons from organellar and nuclear genomes. Nat. Commun..

[10] Leebens-Mack J.H., Barker M.S., Carpenter E.J., Deyholos M.K., Gitzendanner M.A., Graham S.W., Grosse I., Li Z., Melkonian M., Mirarab S. (2019). One thousand plant transcriptomes and the phylogenomics of green plants. Nature.

[11] Degnan J.H., Rosenberg N.A. (2009). Gene tree discordance, phylogenetic inference and the multispecies coalescent. Trends Ecol. Evol..

[12] Soltis D.E., Albert V.A., Leebens-Mack J., Bell C.D., Paterson A.H., Zheng C., Sankoff D., de Pamphilis C.W., Wall P.K., Soltis P.S. (2009). Polyploidy and angiosperm diversification. Am. J. Bot..

[13] Van de Peer Y., Mizrachi E., Marchal K. (2017). The evolutionary significance of polyploidy. Nat. Rev. Genet..

[14] Clark J.W., Donoghue P.C.J. (2018). Whole-genome duplication and plant macroevolution. Trends Plant Sci..

[15] Cai L., Xi Z., Amorim A.M., Sugumaran M., Rest J.S., Liu L., Davis C.C. (2019). Widespread ancient whole-genome duplications in Malpighiales coincide with Eocene global climatic upheaval. New Phytol..

[16] Wood T.E., Takebayashi N., Barker M.S., Mayrose I., Greenspoon P.B., Rieseberg L.H. (2009). The frequency of polyploid speciation in vascular plants. Proc. Natl. Acad. Sci. USA.

[17] Ren R., Wang H., Guo C., Zhang N., Zeng L., Chen Y., Ma H., Qi J. (2018). Widespread whole genome duplications contribute to genome complexity and species diversity in angiosperms. Mol. Plant.

[18] Jiao Y. (2018). Double the genome, double the fun: Genome duplications in angiosperms. Mol. Plant.

[19] Shen H., Jin D., Shu J.-P., Zhou X.-L., Lei M., Wei R., Shang H., Wei H.-J., Zhang R., Liu L. (2018). Large-scale phylogenomic analysis resolves a backbone phylogeny in ferns. GigaScience.

[20] PPGI (2016). A community-derived classification for extant lycophytes and ferns. J. Syst. Evol..

[21] Du X.Y., Lu J.M., Zhang L.B., Wen J., Kuo L.Y., Mynssen C.M., Schneider H., Li D.Z. (2021). Simultaneous diversification of Polypodiales and angiosperms in the Mesozoic. Cladistics.

[22] Qi X., Kuo L.-Y., Guo C., Li H., Li Z., Qi J., Wang L., Hu Y., Xiang J., Zhang C. (2018). A well-resolved fern nuclear phylogeny reveals the evolution history of numerous transcription factor families. Mol. Phylogenet. Evol..

[23] Testo W., Sundue M. (2016). A 4000-species dataset provides new insight into the evolution of ferns. Mol. Phylogenet. Evol..

[24] Schneider H., Schuettpelz E., Pryer K.M., Cranfill R., Magallón S., Lupia R. (2004). Ferns diversified in the shadow of angiosperms. Nature.

[25] Pryer K.M., Schneider H., Smith A.R., Cranfill R., Wolf P.G., Hunt J.S., Sipes S.D. (2001). Horsetails and ferns are a monophyletic group and the closest living relatives to seed plants. Nature.

[26] Rothfels C.J., Li F.-W., Sigel E.M., Huiet L., Larsson A., Burge D.O., Ruhsam M., Deyholos M., Soltis D.E., Stewart C.N. (2015). The evolutionary history of ferns inferred from 25 low-copy nuclear genes. Am. J. Bot..

[27] Kuo L.-Y., Qi X., Ma H., Li F.-W. (2018). Order-level fern plastome phylogenomics: New insights from Hymenophyllales. Am. J. Bot..

[28] Schuettpelz E., Pryer K.M. (2007). Fern phylogeny inferred from 400 leptosporangiate species and three plastid genes. Taxon.

[29] Doweld A. (2001). Prosyllabus Tracheophytorum: Tentamen Systematis Plantarum Vascularium (Tracheophyta).

[30] Reveal J. (1993). New ordinal names for extant vascular plants. Phytologia.

[31] Philippe H., Vienne D.M., Ranwez V., Roure B., Baurain D., Delsuc F. (2017). Pitfalls in supermatrix phylogenomics. Eur. J. Taxon..

[32] Jeffroy O., Brinkmann H., Delsuc F., Philippe H. (2006). Phylogenomics: The beginning of incongruence?. Trends Genet..

[33] Heath T.A., Hedtke S.M., Hillis D.M. (2008). Taxon sampling and the accuracy of phylogenetic analyses. J. Syst. Evol..

[34] Pick K., Philippe H., Schreiber F., Erpenbeck D., Jackson D., Wrede P., Wiens M., Alié A., Morgenstern B., Manuel M. (2010). Improved phylogenomic taxon sampling noticeably affects nonbilaterian relationships. Mol. Biol. Evol..

[35] Nabhan A.R., Sarkar I.N. (2012). The impact of taxon sampling on phylogenetic inference: A review of two decades of controversy. Brief Bioinform..

[36] Qiu Y.-L. (2018). Phylogeny and evolution of vascular plants. eLS.

[37] Haufler C.H. (2014). Ever since Klekowski: Testing a set of radical hypotheses revives the genetics of ferns and lycophytes. Am. J. Bot..

[38] Clark J., Hidalgo O., Pellicer J., Liu H., Marquardt J., Robert Y., Christenhusz M., Zhang S., Gibby M., Leitch I.J. (2016). Genome evolution of ferns: Evidence for relative stasis of genome size across the fern phylogeny. New Phytol..

[39] Huang C.H., Qi X., Chen D., Qi J., Ma H. (2020). Recurrent genome duplication events likely contributed to both the ancient and recent rise of ferns. J. Integr. Plant Biol..

[40] Otto S.P. (2007). The evolutionary consequences of polyploidy. Cell.

[41] Soltis P.S., Marchant D.B., Van de Peer Y., Soltis D.E. (2015). Polyploidy and genome evolution in plants. Curr. Opin. Genet. Dev..

[42] Mayrose I., Zhan S.H., Rothfels C.J., Arrigo N., Barker M.S., Rieseberg L.H., Otto S.P. (2015). Methods for studying polyploid diversification and the dead end hypothesis: A reply to Soltis et al., (2014). New Phytol..

[43] Arrigo N., Barker M.S. (2012). Rarely successful polyploids and their legacy in plant genomes. Curr. Opin. Plant Biol..

[44] Mayrose I., Zhan S.H., Rothfels C.J., Magnuson-Ford K., Barker M.S., Rieseberg L.H., Otto S.P. (2011). Recently formed polyploid plants diversify at lower rates. Science.

[45] Schranz M.E., Mohammadin S., Edger P.P. (2012). Ancient whole genome duplications, novelty and diversification: The WGD Radiation Lag-Time Model. Curr. Opin. Plant Biol..

[46] Tank D.C., Eastman J.M., Pennell M.W., Soltis P.S., Soltis D.E., Hinchliff C.E., Brown J.W., Sessa E.B., Harmon L.J. (2015). Nested radiations and the pulse of angiosperm diversification: Increased diversification rates often follow whole genome duplications. New Phytol..

[47] Meier J.I., Marques D.A., Mwaiko S., Wagner C.E., Excoffier L., Seehausen O. (2017). Ancient hybridization fuels rapid cichlid fish adaptive radiations. Nat. Commun..

[48] Morrison D.A. (2014). Phylogenetic networks: A review of methods to display evolutionary history. Annu. Res. Rev. Biol..

[49] Chao D.-Y., Dilkes B., Luo H., Douglas A., Yakubova E., Lahner B., Salt D.E. (2013). Polyploids exhibit higher potassium uptake and salinity tolerance in Arabidopsis. Science.

[50] Ramsey J. (2011). Polyploidy and ecological adaptation in wild yarrow. Proc. Natl. Acad. Sci. USA.

[51] Diallo A.M., Nielsen L.R., Kjær E.D., Petersen K.K., Ræbild A. (2016). Polyploidy can confer superiority to West African *Acacia senegal* (L.) Willd. trees. Front. Plant Sci..

[52] Fawcett J.A., Maere S., Van De Peer Y. (2009). Plants with double genomes might have had a better chance to survive the Cretaceous–Tertiary extinction event. Proc. Natl. Acad. Sci. USA.

[53] Vanneste K., Baele G., Maere S., Van de Peer Y. (2014). Analysis of 41 plant genomes supports a wave of successful genome duplications in association with the Cretaceous–Paleogene boundary. Genome Res..

[54] Wang Y., Yang X., Guignard G., Deng S., Tian N., Jiang Z. (2009). The fossil Gleicheniaceous ferns of China: Biodiversity, systematics, spore ultrastructure and evolution. Rev. Palaeobot. Palynol..

[55] Clapham M.E., Renne P.R. (2019). Flood basalts and mass extinctions. Annu. Rev. Earth Planet. Sci..

[56] Sun H., Xiao Y., Gao Y., Zhang G., Casey J.F., Shen Y. (2018). Rapid enhancement of chemical weathering recorded by extremely light seawater lithium isotopes at the Permian–Triassic boundary. Proc. Natl. Acad. Sci. USA.

[57] Corsin P. Paleobiogeography of the dipteridaceae and matoniaceae of the mesozoic. Proceedings of the IV International Gondwana Symposium.

[58] Wang Y. (2002). Fern ecological implications from the Lower Jurassic in western Hubei, China. Rev. Palaeobot. Palynol..

[59] Pryer K.M., Schuettpelz E., Wolf P.G., Schneider H., Smith A.R., Cranfill R. (2004). Phylogeny and evolution of ferns (monilophytes) with a focus on the early leptosporangiate divergences. Am. J. Bot..

[60] Kramer K.U., Green P., Kubitzki K. (1990). The Families and Genera of Vascular Plants. V. 1: Pteridophytes and Gymnosperms.

[61] Schuettpelz E., Korall P., Pryer K.M. (2006). Plastid atpA data provide improved support for deep relationships among ferns. Taxon.

[62] Schneider H. (1996). Vergleichende Wurzelanatomie der Farne.

[63] Smith A.R., Pryer K.M., Schuettpelz E., Korall P., Schneider H., Wolf P.G. (2006). A classification for extant ferns. Taxon.

[64] Bierhorst D.W. (1971). Morphology of Vascular Plants.

[65] Tryon A.F., Lugardon B. (2012). Spores of the Pteridophyta: Surface, Wall Structure, and Diversity Based on Electron Microscope Studies.

[66] Holttum R.E. (1953). A Revised Flora of Malaya: Ferns of Malaya.

[67] Zhang X., Masahiro K., Hans P.N., Wu Z.Y., Raven P.H., Hong D.Y. (2013). Dipteridaceae. Flora of China.

[68] Turland N.J., Wiersema J.H., Barrie F.R., Greuter W., Hawksworth D.L., Herendeen P.S., Knapp S., Kusber W.-H., Li D.-Z., Marhold K. (2018). International Code of Nomenclature for Algae, Fungi, and Plants (Shenzhen Code) Adopted by the Nineteenth International Botanical Congress Shenzhen, China, July 2017.

[69] Skog J.E. (2001). Biogeography of Mesozoic leptosporangiate ferns related to extant ferns. Brittonia.

[70] Taylor E.L., Taylor T.N., Krings M. (2009). Paleobotany: The Biology and Evolution of Fossil Plants.

[71] Zhou N., Wang Y.-D., Li L.-Q., Zhang X.-Q. (2016). Diversity variation and tempo-spatial distributions of the Dipteridaceae ferns in the Mesozoic of China. Palaeoworld.

[72] Choo T.Y., Escapa I.H. (2018). Assessing the evolutionary history of the fern family Dipteridaceae (Gleicheniales) by incorporating both extant and extinct members in a combined phylogenetic study. Am. J. Bot..

[73] Wang Y., Zhang H. (2010). Fertile organs and in situ spores of a new dipteridaceous fern *Hausmannia sinensis* from the Jurassic of northern China. Proc. R. Soc. B Biol. Sci..

[74] Wang Y., Li L., Guignard G., Dilcher D.L., Xie X., Tian N., Zhou N., Wang Y. (2015). Fertile structures with in situ spores of a dipterid fern from the Triassic in southern China. J. Plant Res..

[75] Guignard G., Wang Y., Ni Q., Tian N., Jiang Z. (2009). A dipteridaceous fern with in situ spores from the Lower Jurassic in Hubei, China. Rev. Palaeobot. Palynol..

[76] Chase M.W., Christenhusz M., Fay M., Byng J., Judd W.S., Soltis D., Mabberley D., Sennikov A., Soltis P.S., Stevens P.F. (2016). An update of the Angiosperm Phylogeny Group classification for the orders and families of flowering plants: APG IV. Bot. J. Linn. Soc..

[77] Klass K.-D., Zompro O., Kristensen N.P., Adis J. (2002). Mantophasmatodea: A new insect order with extant members in the Afrotropics. Science.

[78] Shu J.-P., Shang H., Jin D., Wei H.-J., Zhou X.-L., Liu H.-M., Gu Y.-F., Wang Y., Wang F.-G., Shen H. (2017). Re-establishment of species from synonymies based on DNA barcoding and phylogenetic analysis using *Diplopterygium simulans* (Gleicheniaceae) as an example. PLoS ONE.

[79] Simão F.A., Waterhouse R.M., Ioannidis P., Kriventseva E.V., Zdobnov E.M. (2015). BUSCO: Assessing genome assembly and annotation completeness with single-copy orthologs. Bioinformatics.

[80] Douard V., Brunet F., Boussau B., Ahrens-Fath I., Vlaeminck-Guillem V., Haendler B., Laudet V., Guiguen Y. (2008). The fate of the duplicated androgen receptor in fishes: A late neofunctionalization event?. BMC Evol. Biol..

[81] Dunn C.W., Howison M., Zapata F. (2013). Agalma: An automated phylogenomics workflow. BMC Bioinform..

[82] Breinholt J.W., Kawahara A.Y. (2013). Phylotranscriptomics: Saturated third codon positions radically influence the estimation of trees based on next-gen data. Genome Biol. Evol..

[83] Ran J.-H., Shen T.-T., Wang M.-M., Wang X.-Q. (2018). Phylogenomics resolves the deep phylogeny of seed plants and indicates partial convergent or homoplastic evolution between Gnetales and angiosperms. Proc. R. Soc. B Biol. Sci..

[84] Stamatakis A. (2014). RAxML version 8: A tool for phylogenetic analysis and post-analysis of large phylogenies. Bioinformatics.

[85] Mirarab S., Reaz R., Bayzid M.S., Zimmermann T., Swenson M.S., Warnow T. (2014). ASTRAL: Genome-scale coalescent-based species tree estimation. Bioinformatics.

[86] Seo T.-K. (2008). Calculating bootstrap probabilities of phylogeny using multilocus sequence data. Mol. Biol. Evol..

[87] Huson D.H., Bryant D. (2006). Application of phylogenetic networks in evolutionary studies. Mol. Biol. Evol..

[88] Bryant D., Moulton V. (2004). Neighbor-net: An agglomerative method for the construction of phylogenetic networks. Mol. Biol. Evol..

[89] Bek J., Pšenička J. (2001). *Senftenbergia plumosa* (Artis) emend. and its spores from the Carboniferous of the Kladno and Pilsen basins, Bohemian Massif, and some related and synonymous taxa. Rev. Palaeobot. Palynol..

[90] Axsmith B.J., Krings M., Taylor T.N. (2001). A filmy fern from the Upper Triassic of North Carolina (USA). Am. J. Bot..

[91] Yang Z. (2007). PAML 4: Phylogenetic analysis by maximum likelihood. Mol. Biol. Evol..

[92] Tiley G.P., Barker M.S., Burleigh J.G. (2018). Assessing the Performance of Ks Plots for Detecting Ancient Whole Genome Duplications. Genome Biol. Evol..

[93] Conant G.C., Birchler J.A., Pires J.C. (2014). Dosage, duplication, and diploidization: Clarifying the interplay of multiple models for duplicate gene evolution over time. Curr. Opin. Plant Biol..

[94] Zwaenepoel A., Van de Peer Y. (2019). Inference of Ancient Whole-Genome Duplications and the Evolution of Gene Duplication and Loss Rates. Mol. Biol. Evol..

[95] Paterson A., Bowers J., Chapman B. (2004). Ancient polyploidization predating divergence of the cereals, and its consequences for comparative genomics. Proc. Natl. Acad. Sci. USA.

[96] Li Z., Baniaga A.E., Sessa E.B., Scascitelli M., Graham S.W., Rieseberg L.H., Barker M.S. (2015). Early genome duplications in conifers and other seed plants. Sci. Adv..

[97] Maliet O., Hartig F., Morlon H. (2019). A model with many small shifts for estimating species-specific diversification rates. Nat. Ecol. Evol..

[98] Stadler T. (2019). Species-specific diversification. Nat. Ecol. Evol..

