# Supplementary material for: Phylogenomic Analysis Reconstructed the Order Matoniales from Paleopolyploidy Veil
Source: Plants (Basel). 2022 Jun 7;11(12):1529. doi: 10.3390/plants11121529 (PMC9228301; doi:10.3390/plants11121529)
Supplement: Supplementary file 1 [file plants-11-01529-s001.zip › plants-1724612-supplementary-figures.pdf]

Supplementary Material

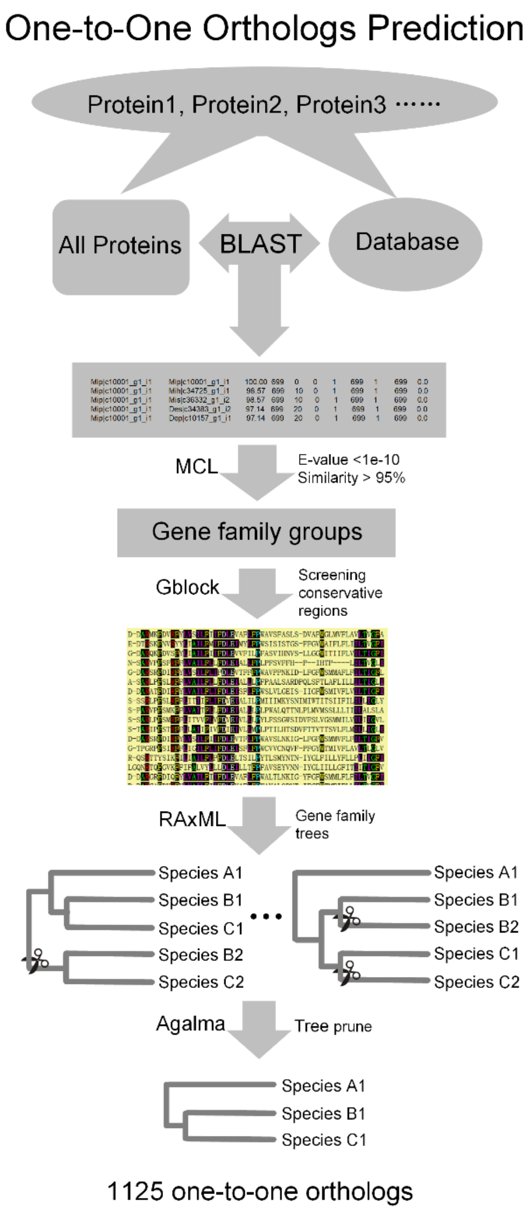

Figure S1. The pipeline of 1-to-1 orthologs prediction.

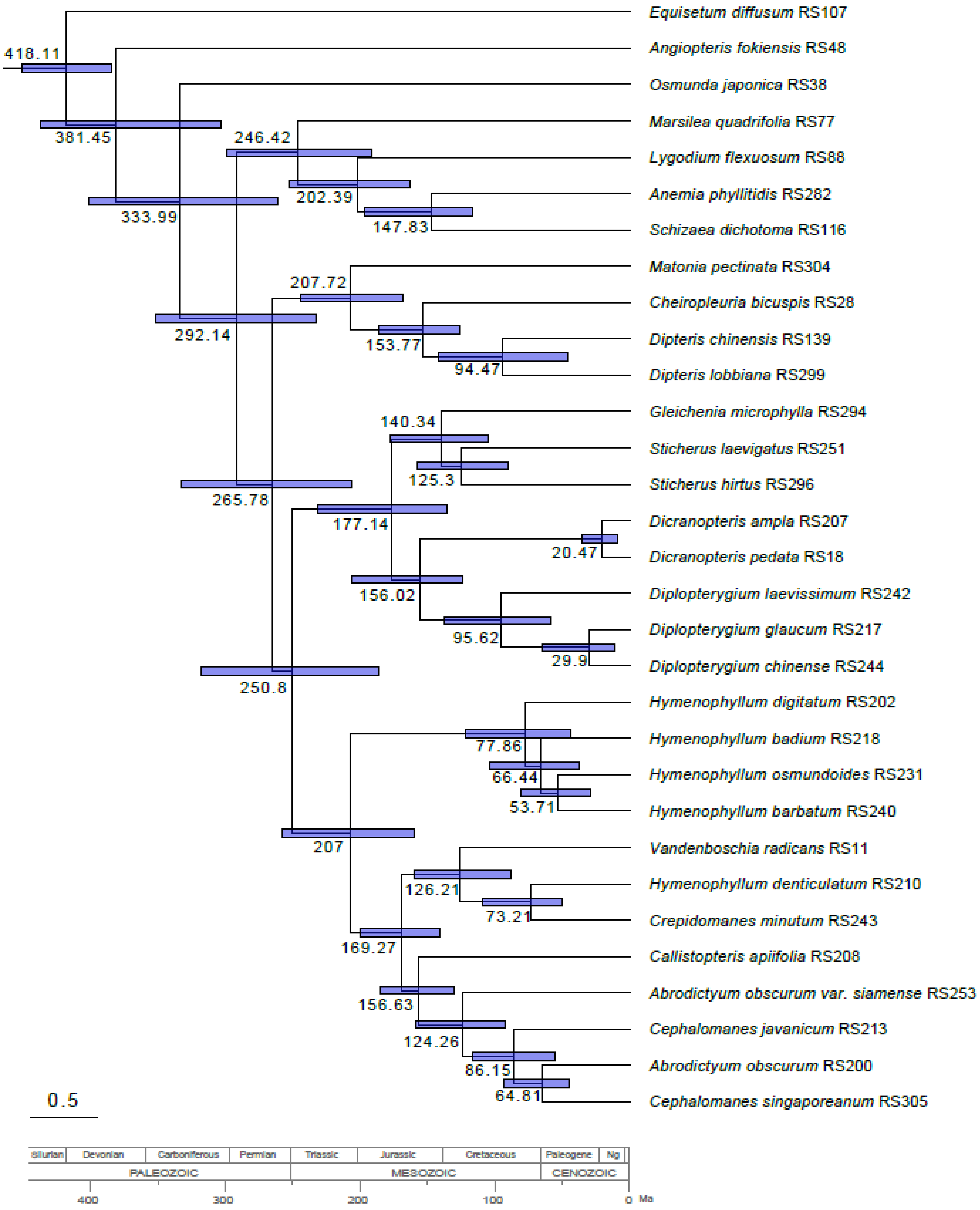

Figure S2. The timetree of early leptosporangiate ferns

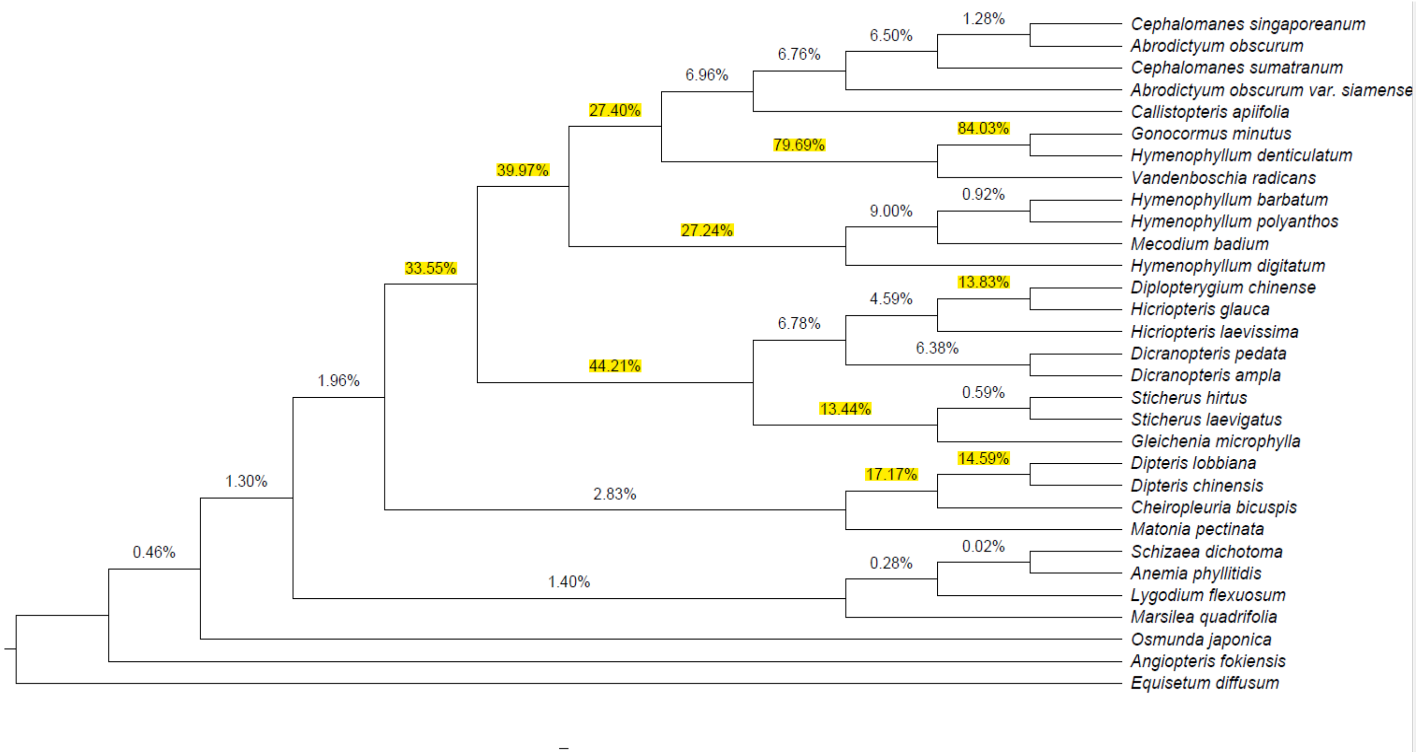

Figure S3. The inference of WGD events in early leptosporangiate ferns.

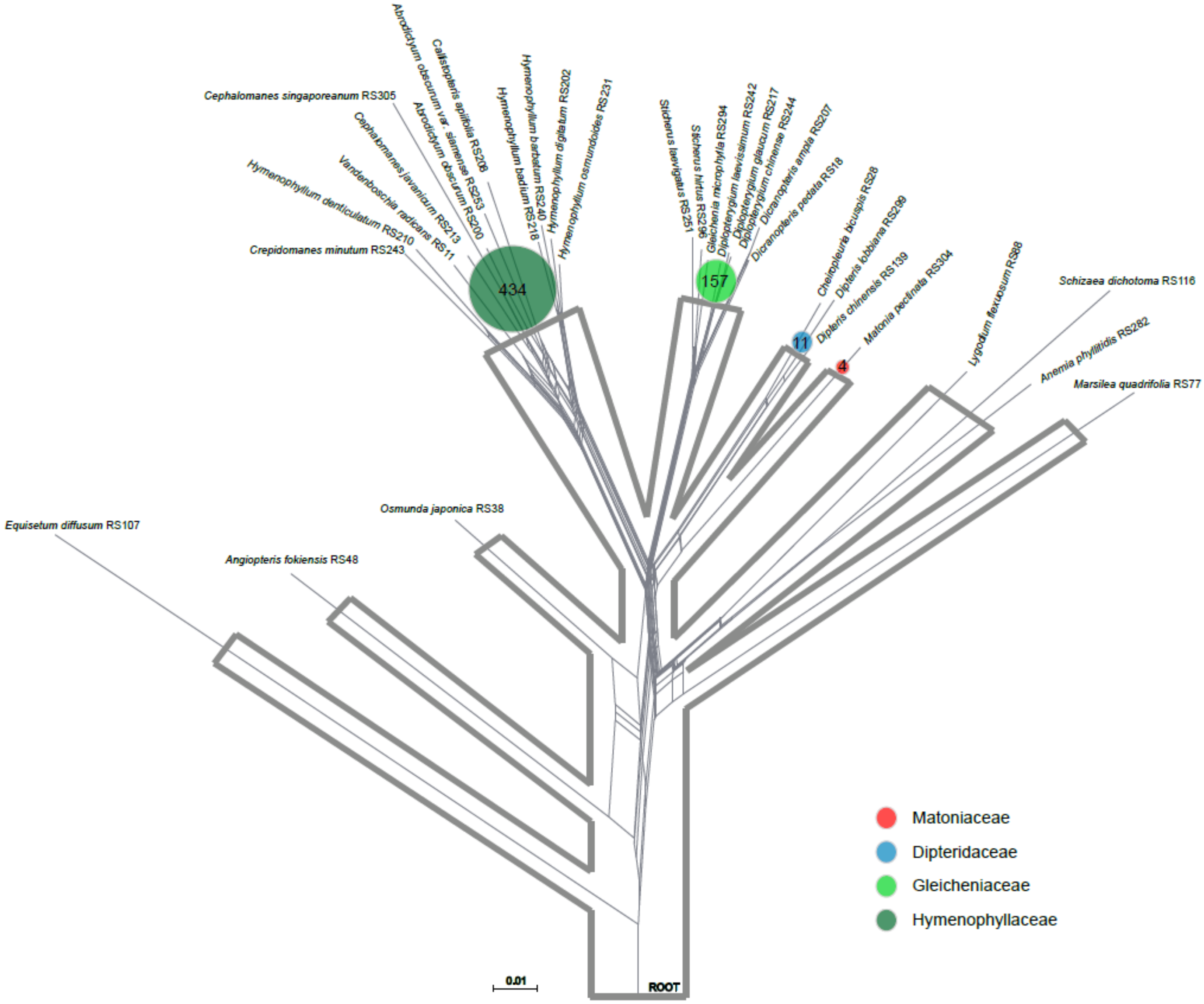

Figure S4. The rooted reticulate evolution in the early leptosporangiate ferns
