# Supplementary material for: Phylogenomic Analysis Reconstructed the Order Matoniales from Paleopolyploidy Veil
Source: Plants (Basel). 2022 Jun 7;11(12):1529. doi: 10.3390/plants11121529 (PMC9228301; doi:10.3390/plants11121529)
Supplement: Supplementary file 1 [file plants-11-01529-s001.zip › Table S1.pdf]

Table S1 Summary of main studies of the basal leptosporangiate ferns. (Gle: Gleicheniales; Hym: Hymenophyllales)

Study	Phylogenetic depth	Sampled			Data types	Methods	Topology
		Ferns	Gle	Hym			
Hasebe et al. (1994)	Leptosporangiate ferns	58	5	3	Plastid (rbcl)	NJ; MP	Fig. 1A
Hasece et al. (1995)	All ferns	107	6	6	Plastid (rbcl)	NJ; MP; ML	Fig. 1D
Pryer et al. (1995)	All ferns	50	5	1	Plastid (rbcl); Morphology (77)	MP	Fig. 1D
Pryer et al. (2001)	Vascular plants	21	2	1	Plastid (3); Nuclear (18S rDNA); Morphology (136)	MP; ML	Fig. 1A
Pryer et al. (2004)	All ferns	53	10	2	Plastid (3); Nuclear (18S rDNA)	MP; ML; BI	Fig. 1C
Schneider et al. (2004)	All ferns	41	1	1	Plastid (2)	BI	Fig. 1A
Wikström & Pryer (2005)	All ferns	20	2	1	Plastid (3); Nuclear (18S rDNA); Mitochondrion (atp1); Morphology (138)	ML; BI	Fig. 1A
Qiu et al. (2006)	Land plants	36	1	2	Plastid (4); Nuclear (18S rDNA); Mitochondrion (LSU r DNA); cp genome (67); Genomic structure (28)	MP; ML	Fig. 1A
Schuettpelz et al. (2006)	All ferns	52	9	2	Plastid (4); Nuclear (18S rDNA)	ML; BI	Fig. 1A
Schuettpelz & Pryer (2006)	All ferns	101	10	50	Plastid (rbcl)	BI	Fig. 1A
Qiu et al. (2007)	Land plants	36	1	2	Plastid (4); Nuclear (18S rDNA); Mitochondrion (2)	ML	Fig. 1A
Schuettpelz & Pryer (2007)	Leptosporangiate ferns	400	11	28	Plastid (3)	ML	Fig. 1A
Rai & Graham (2010)	Land plants	34	4	3	Plastid (17)	MP; ML	Fig. 1A
Kuo et al. (2011)	All ferns	78	3	2	Plastid (3)	ML; BI	Fig. 1F
Lehtonen (2011)	All ferns	2656	35	168	Plastid (4)	ML	Fig. 1A
Knie et al. (2015)	All ferns	30	2	2	Plastid (5); Mitochondrion (4)	ML	Fig. 1B
Rothfels et al. (2015)	All ferns	73	3	2	Nuclear (25)	ML; BI; coalescent	Fig. 1D
Testo et al. (2016)	All ferns	3973	41	209	Plastid (7)	ML	Fig. 1A
Shen et al. (2018)	All ferns	69	2	1	Nuclear (2391/1334)	ML; coalescent	Fig. 1E
Kuo et al. (2018)	All ferns	26	2	2	Plastid (71)	ML	Fig. 1A
Qi et al. (2018)	All ferns	127	8	6	Nuclear (146)	ML; coalescent	Fig. 1E
Wolf et al. (2018)	All ferns	24	1	1	Nuclear (25)	ML	Fig. 1A

Ref.

Hasebe, M., T. Omori, M. Nakazawa, T. Sano, M. Kato and K. Iwatsuki (1994). "rbcL gene sequences provide evidence for the evolutionary lineages of leptosporangiate ferns." Proceedings of the National Academy of Sciences **91**(12): 5730-5734.

Hasebe, M., P. G. Wolf, K. M. Pryer, K. Ueda, M. Ito, R. Sano, G. J. Gastony, J. Yokoyama, J. R. Manhart and N. Murakami (1995). "Fern phylogeny based on rbcL nucleotide sequences." American Fern Journal: 134-181.

Knie, N., S. Fischer, F. Grewe, M. Polsakiewicz and V. Knoop (2015). "Horsetails are the sister group to all other monilophytes and Marattiales are sister to leptosporangiate ferns." Molecular Phylogenetics and Evolution **90**: 140-149.

Kuo, L.-Y., F.-W. Li, W.-L. Chiou and C.-N. Wang (2011). "First insights into fern matK phylogeny." Molecular Phylogenetics and Evolution **59**(3): 556-566.

Kuo, L. Y., X. Qi, H. Ma and F. W. Li (2018). "Order-level fern plastome phylogenomics: new insights from Hymenophyllales." American journal of botany **105**(9): 1545-1555.

Lehtonen, S. (2011). "Towards resolving the complete fern tree of life." PLoS One **6**(10): e24851.

Pryer, K. M., H. Schneider, A. R. Smith, R. Cranfill, P. G. Wolf, J. S. Hunt and S. D. Sipes (2001). "Horsetails and ferns are a monophyletic group and the closest living relatives to seed plants." Nature **409**(6820): 618.

Pryer, K. M., E. Schuettpelz, P. G. Wolf, H. Schneider, A. R. Smith and R. Cranfill (2004). "Phylogeny and evolution of ferns (monilophytes) with a focus on the early leptosporangiate divergences." American journal of Botany **91**(10): 1582-1598.

Pryer, K. M., A. R. Smith and J. E. Skog (1995). "Phylogenetic relationships of extant ferns based on evidence from morphology and rbcL sequences." American Fern Journal: 205-282.

Qi, X., L.-Y. Kuo, C. Guo, H. Li, Z. Li, J. Qi, L. Wang, Y. Hu, J. Xiang and C. Zhang (2018). "A well-resolved fern nuclear phylogeny reveals the evolution history of numerous transcription factor families." Molecular phylogenetics and evolution **127**: 961-977.

Qiu, Y.-L., L. Li, B. Wang, Z. Chen, O. Dombrovska, J. Lee, L. Kent, R. Li, R. W. Jobson and T. A. Hendry (2007). "A nonflowering land plant phylogeny inferred from nucleotide sequences of seven chloroplast, mitochondrial, and nuclear genes." International Journal of Plant Sciences **168**(5): 691-708.

Qiu, Y.-L., L. Li, B. Wang, Z. Chen, V. Knoop, M. Groth-Malonek, O. Dombrovska, J. Lee, L. Kent and J. Rest (2006). "The deepest divergences in land plants inferred from phylogenomic evidence." Proceedings of the National Academy of Sciences **103**(42): 15511-15516.

Rai, H. S. and S. W. Graham (2010). "Utility of a large, multigene plastid data set in inferring higher-order relationships in ferns and relatives (monilophytes)." American Journal of Botany **97**(9): 1444-1456.

Rothfels, C. J., F. W. Li, E. M. Sigel, L. Huiet, A. Larsson, D. O. Burge, M. Ruhsam, M. Deyholos, D. E. Soltis and C. N. Stewart Jr (2015). "The evolutionary history of ferns inferred from 25 low-copy nuclear genes." American Journal of Botany **102**(7): 1089-1107.

Schneider, H., E. Schuettpelz, K. M. Pryer, R. Cranfill, S. Magallón and R. Lupia (2004). "Ferns diversified in the shadow of angiosperms." Nature **428**(6982): 553.

Schuettpelz, E., P. Korall and K. M. Pryer (2006). "Plastid atpA data provide improved support for deep relationships among ferns." Taxon **55**(4): 897-906.

Schuettpelz, E. and K. M. Pryer (2006). "Reconciling extreme branch length differences: decoupling time and rate through the evolutionary history of filmy ferns." Systematic Biology **55**(3): 485-502.

Schuettpelz, E. and K. M. Pryer (2007). "Fern phylogeny inferred from 400 leptosporangiate species and three plastid genes." Taxon **56**(4): 1037-1050.

Shen, H., D. Jin, J.-P. Shu, X.-L. Zhou, M. Lei, R. Wei, H. Shang, H.-J. Wei, R. Zhang, L. Liu, Y.-F. Gu, X.-C. Zhang and Y.-H. Yan (2017). "Large-scale phylogenomic analysis resolves a backbone phylogeny in ferns." GigaScience **7**(2).

Testo, W. and M. Sundue (2016). "A 4000-species dataset provides new insight into the evolution of ferns." Molecular Phylogenetics and Evolution **105**: 200-211.

Wikström, N. and K. M. Pryer (2005). "Incongruence between primary sequence data and the distribution of a mitochondrial atp1 group II intron among ferns and horsetails." Molecular Phylogenetics and Evolution

36(3): 484-493.

Wolf, P. G., T. A. Robison, M. G. Johnson, M. A. Sundue, W. L. Testo and C. J. Rothfels (2018). "Target sequence capture of nuclear-encoded genes for phylogenetic analysis in ferns." Applications in plant sciences **6**(5): e01148.
