# Supplementary material for: Phylogenomic Analysis Reconstructed the Order Matoniales from Paleopolyploidy Veil
Source: Plants (Basel). 2022 Jun 7;11(12):1529. doi: 10.3390/plants11121529 (PMC9228301; doi:10.3390/plants11121529)
Supplement: Supplementary file 1 [file plants-11-01529-s001.zip › Table S2.pdf]

Table S2. The informations of samples and transcriptome data used in this study.

Samples ID	Species	Family	Clean data (G)	Total reads (clean)	Q30 (%)	Number of contigs	N50 (bp)	Mean (bp)
RS11	*Vandenboschia radicans*	Hymenophyllacea	4.8	38639790	90.3	273743	625	486.12
RS18	*Dicranopteris pedata*	Gleicheniaceae	4.2	33942120	92.04	75259	1435	829.01
RS28	*Cheiropleuria bicuspis*	Dipteridaceae	5.1	41617294	91.35	143200	1954	1182.58
RS38	*Osmunda japonica*	Osmundaceae	4.1	33485274	92.05	66318	2109	1163.17
RS48	*Angiopteris fokiensis*	Angiopteridaceae	4.4	35120302	91.12	63003	1904	1188.75
RS77	*Marsilea quadrifolia*	Marsileaceae	4.3	34724432	91.76	85761	2045	1311.10
RS88	*Lygodium flexuosum*	Lygodiaceae	4.2	34098316	91.44	94774	2003	1253.49
RS107	*Equisetum diffusum*	Equisetaceae	4.4	35693238	90.21	117880	1704	908.40
RS116	*Schizaea dichotoma*	Schizaeaceae	4.5	36668734	89.6	70687	1585	1018.15
RS139	*Dipteris chinensis*	Dipteridaceae	9.4	63997114	94.65	126520	2117	1151.61
RS200	*Abrodictyum obscurum*	Hymenophyllacea	9.3	63065130	94.77	119181	2273	1182.73
RS202	*Hymenophyllum digitatum*	Hymenophyllacea	8.0	54129212	95.41	184930	867	553.72
RS207	*Dicranopteris ampla*	Gleicheniaceae	9.6	65013104	95.65	76029	1521	975.60
RS208	*Callistopteris apiifolia*	Hymenophyllacea	8.5	58206276	95.27	685642	435	438.86
RS210	*Hymenophyllum denticulatum*	Hymenophyllacea	9.1	61421476	95.18	124284	2144	1247.54
RS213	*Cephalomanes javanicum*	Hymenophyllacea	8.1	54990600	95.07	92514	2196	1192.94
RS217	*Diplopterygium glaucum*	Gleicheniaceae	10.8	73301566	94.98	255509	1682	817.10
RS218	*Hymenophyllum badium*	Hymenophyllacea	9.2	62303336	94.87	256540	1697	895.66
RS231	*Hymenophyllum osmundoides*	Hymenophyllacea	10.0	67318452	95.54	161621	1618	795.13
RS240	*Hymenophyllum barbatum*	Hymenophyllacea	9.0	60903248	95.14	257884	1536	759.24
RS242	*Diplopterygium laevissimum*	Gleicheniaceae	9.5	64492260	95.05	107038	2177	1278.81
RS243	*Crepidomanes minutum*	Hymenophyllacea	9.2	61799022	95.47	183181	1882	927.47
RS244	*Diplopterygium chinense*	Gleicheniaceae	10.4	70702896	95.29	164858	2021	1022.95
RS251	*Sticherus laevigatus*	Gleicheniaceae	8.6	58197742	95.97	154701	2277	1240.72
RS253	*Abrodictyum obscurum var. siamense*	Hymenophyllacea	7.5	51437264	91.96	98928	2119	1057.03
RS282	*Anemia phyllitidis*	Anemiaceae	7.4	49991062	95.86	186139	1813	900.36
RS294	*Gleichenia microphylla*	Gleicheniaceae	15.5	104739352	88.79	150169	1863	941.13
RS296	*Sticherus hirtus*	Gleicheniaceae	12.1	81606118	90.42	107200	2173	1274.65
RS299	*Dipteris lobbiana*	Dipteridaceae	12.9	86315908	92.33	391485	1461	790.88
RS304	*Matonia pectinata*	Matoniaceae	8.3	55132016	90.84	162856	2060	1023.26
RS305	*Cephalomanes singaporeanum*	Hymenophyllacea	7.4	49190052	91.24	634401	746	568.78
